# Tumor Size Measurements for Predicting Hodgkin’s and Non-Hodgkin’s Lymphoma Response to Treatment

**DOI:** 10.3390/metabo12040285

**Published:** 2022-03-24

**Authors:** Maria Kallergi, Alexandros Georgakopoulos, Vassiliki Lyra, Sofia Chatziioannou

**Affiliations:** 1 Department of Biomedical Engineering, University of West Attica, 12243 Athens, Greece; 2 Division of Nuclear Medicine, Biomedical Research Foundation of the Academy of Athens, 11527 Athens, Greece; ageorgakopoulos@bioacademy.gr (A.G.); sofiac@med.uoa.gr (S.C.); 3 2nd Department of Radiology, Nuclear Medicine Section, Attikon University Hospital of Athens, 12462 Chaidari, Greece; 4 Nuclear Medicine Department, General University Hospital of Larissa, 41110 Larissa, Greece; vassilikilyra@gmail.com

**Keywords:** metabolic tumor size, metabolic volume, PET/CT, lymphoma, image segmentation

## Abstract

The purpose of this study was to investigate the value of tumor size measurements as prognostic indicators of treatment outcome of Hodgkin’s and Non-Hodgkin’s lymphomas. ^18^F-FDG PET/CT exams before and after treatment were analyzed and metabolic and anatomic parameters—tumor maximum diameter, tumor maximum area, tumor volume, and maximum standardized uptake value (SUVmax)—were determined manually by an expert and automatically by a computer algorithm on PET and CT images. Results showed that the computer algorithm measurements did not correlate well with the expert’s standard maximum tumor diameter measurements but yielded better three dimensional metrics that could have clinical value. SUVmax was the strongest prognostic indicator of the clinical outcome after treatment, followed by the automated metabolic tumor volume measurements and the expert’s metabolic maximum diameter measurements. Anatomic tumor measurements had poor prognostic value. Metabolic volume measurements, although promising, did not significantly surpass current standard of practice, but automated measurements offered a significant advantage in terms of time and effort and minimized biases and variances in the PET measurements. Overall, considering the limited value of tumor size in predicting response to treatment, a paradigm shift seems necessary in order to identify robust prognostic markers in PET/CT; radiomics, namely combinations of anatomy, metabolism, and imaging, may be an option.

## 1. Introduction

Identifying the right treatment for the oncology patient is paramount to a successful outcome. Determining early on whether a tumor responds or not to a certain treatment followed is paramount to the selection of the right treatment and a good prognosis. There is significant effort in identifying metrics from imaging or other diagnostic studies that could be correlated to outcome and will allow us to predict response based on a tumor’s anatomical or metabolic/functional characteristics. A treatment is effective if clinical symptoms and survival improve and tumor size is reduced. PET/CT hybrid imaging offers both anatomic and functional information and attractive metrics for monitoring tumor response to treatment in one-, two-, and three-dimensions [1]. 

Currently one-dimensional (1D) measurements are the clinical standard. There is significant research effort, however, to demonstrate that the measurement of a single dimension of a tumor is oversimplification; it does not adequately represent its irregular shape, and its often nonsymmetrical changes to a specific treatment or over time. There are numerous reports on the possibly higher value of three-dimensional (3D) tumor size metrics as prognostic indicators of an oncology patient’s response to treatment. However, all studies are coming short of spectacular results when 3D metrics are evaluated, and the standard of practice remains unchanged and includes the metabolic tumor maximum diameter (MTDmax), which is a 1D metric, and SUVmax [2].

We can support the thesis that one type of measurement does not fit all cases. However, we can see evidence that complicated approaches do not necessarily mean better outcomes or better health care. The issue is timely intervention, and if this can be achieved via relatively simple, standardized means, then it is preferable. One should also consider the bias and variances identified in PET/CT tumor measurements in the process of developing a robust and reproducible treatment outcome metric [1]. 

Lymphomas have unique characteristics, different from solid tumors, which have been studied extensively in the last 20 years including the value of metabolic tumor volume (MTV) as a prognostic marker of Hodgkin’s Lymphoma (HL) and Non-Hodgkin’s Lymphoma (NHL) response to treatment [3,4]. Limitations of CT for these patients have been clearly demonstrated [4,5], but PET results are also contradicting; no consensus has been achieved on the value of metabolic parameters, while the initial method of measuring and reporting MTDmax and SUVmax remains a universal practice [5]. It is possible that the large number of variables interferes with the standardization of the measurement and its wider acceptance. So, the question remains: Should we strive for metabolic tumor volume measurements or be content with maximum diameter measurements and try to automate and standardize the latter as much as possible on PET data or look for completely new metrics altogether and re-evaluate the role of CT?

This study aimed at addressing some of the previous questions for HL and NHL patients, who underwent PET/CT before and after treatment and were followed up clinically after the end of their treatment. In a pilot study with HL only, the MTV showed significant correlation with treatment outcome or prognostic value [6]. We expanded our work, however, to include more NHL cases and complete clinical follow up. The new data showed that MTDmax is a reliable, universal, fast, and easy to apply measurement with good prognostic value. In agreement with studies in other pathologies, MTV added some but not significant prognostic value in HL and NHL cases [7,8,9,10]. Manual MTV estimates were time consuming to perform, while an automated approach was more promising and yielded significantly better results than an expert. 

## 2. Results

### 2.1. Patient Characteristics and Measurements

In this study, we expanded our pilot work, results of which were reported in 2015 [6], to include 24 NHL and 21 HL patients with one mass of interest each. The large majority of the NHL patients (about 80%) had diffuse large B-cell lymphoma, while the large majority of HL was nodular sclerosis (about 90%) ^18^F-FDG PET/CT scans of each patient were taken before and after chemotherapy and radiation therapy with the same imaging protocol. The following measurements were performed on the PET images for all masses:

(a) Expert’s PET measurements: Metabolic tumor maximum diameter (MTDmax) and SUVmax from a single transverse PET image, metabolic tumor volume (MTV) from all slices where tumor was present, pre and post treatment.
(b) Computer aided detection (CAD) algorithm PET measurements: MTDmax, metabolic tumor maximum area (MTAmax) from a single transverse PET image, MTV from all slices where tumor was present, pre and post treatment.
(c) CAD CT measurements: Maximum tumor diameter (Dmax), tumor maximum area (Amax) from the single transverse CT image that corresponded to the single PET slice used in the previous measurements, tumor volume (TV) from all CT slices where tumor was present, pre and post treatment.

Average values of the metabolic measurements from the PET images and their standard deviations performed by the expert and the CAD pre and post treatment are listed in Table 1 and Table 2 for the HL and NHL patients, respectively. Table 3 summarizes the CAD CT measurements.

### 2.2. RECIL Classification of Changes in Measured Variables

Changes in the PET variables between pre and post-treatment were calculated by subtracting the baseline PET measurement from the post treatment measurement and dividing by the baseline measurement. The %change was classified in four categories based on the response evaluation criteria in lymphoma (RECIL) [11,12]. Specifically, patients’ response to treatment was distinguished as

i. Complete metabolic response (CMR): Complete disappearance of the lesion; area of tumor is indistinguishable from surrounding tissue (−100% change).
ii. Partial metabolic response (PMR): At least 30% reduction in metabolic tumor size post treatment, i.e., −100% < Δ ≤ −30%.
iii. Stable metabolic disease (SMD): Less than 20% increase or less than 30% decrease in tumor size and no new lesions, i.e., −30% < Δ < 20%.
iv. Progressive metabolic disease (PMD): At least 20% increase in tumor or appearance of new lesions (Δ ≥ 20%).

**Figure 1:**
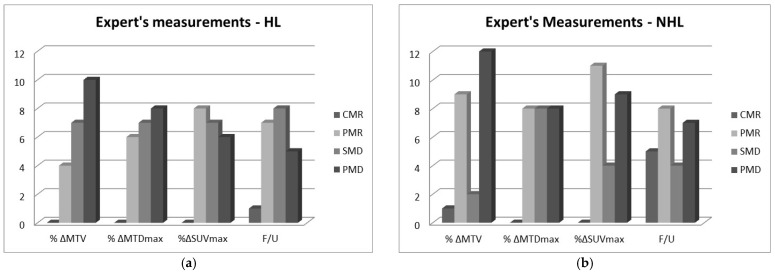
Distribution of the number of RECIL classes estimated from the expert’s measurements of relative percent changes in volume, maximum diameter, and SUV max, i.e., %ΔMTV, %ΔMTDmax, %Δ SUVmax for the (**a**) HL and (**b**) the NHL cases. The follow-up (F/U) clinical classification of the patients is also included.

**Figure 2:**
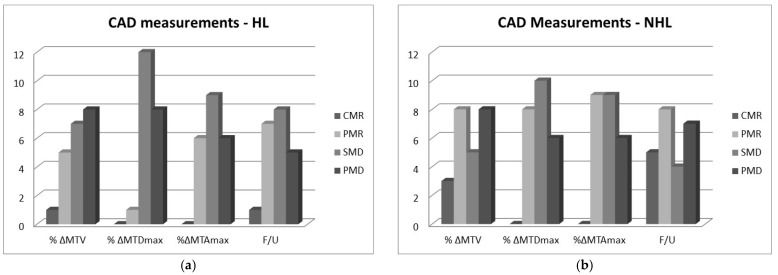
Distribution of the number of RECIL classes estimated from CAD’s measurements of relative percent changes in volume, maximum diameter, and maximum area, i.e., %ΔMTV, %ΔMTDmax, %ΔMTAmax for the (**a**) HL and (**b**) the NHL cases. The follow-up (F/U) clinical classification of the patients is also included.

It should be noted that the numbers 3, 2, 1, and 0 were assigned to the above four classes, respectively, for further analysis. The classification of the patients that was based on the expert’s or CAD’s measurements were compared to the clinical outcome, i.e., the classification of each patient at the first follow-up after the end of the treatment. The distribution of the frequencies of the treatment response classes are shown in Figure 1 and Figure 2. 

### 2.3. Differences between Expert and CAD

**Figure 3:**
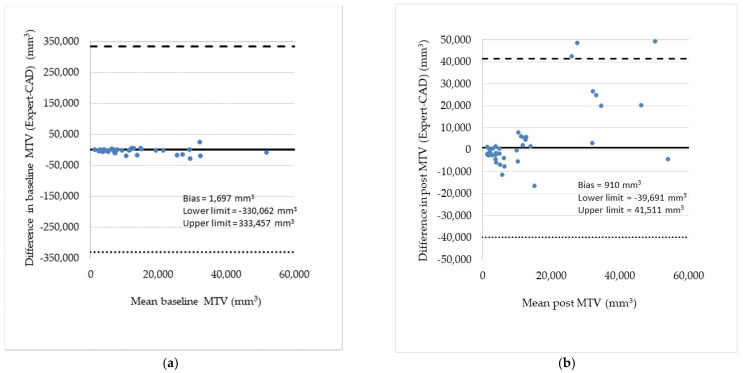
(**a**) Bland–Altman plot showing the differences between expert and CAD baseline measurements of MTV for all patients. Two measurements above the upper limit are omitted for presentation purposes. (**b**) Similar plot for the post treatment measurements of MTV where differences have higher variability. The bias (mean value of the differences) and the lower and upper limits of agreement are shown in the inserts. In both pre and post treatment measurements, the differences and the variability (scatter) tend to increase as the mean MTV increases.

**Figure 4:**
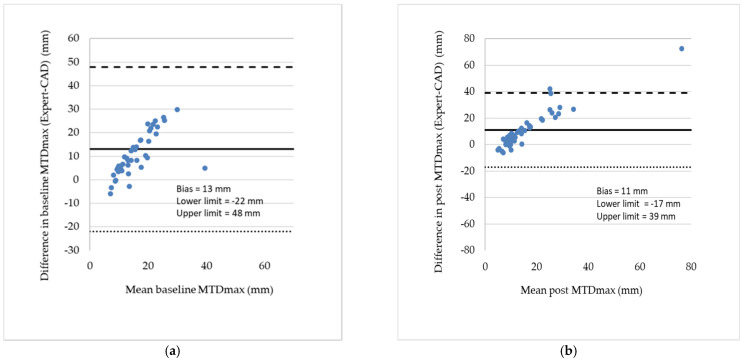
(**a**) Bland–Altman plot showing the differences between expert and CAD baseline measurements of MTDmax for all patients. (**b**) Similar plot for the post treatment measurements of MTDmax. The bias (mean value of the differences) and the lower and upper limits of agreement are shown in the inserts. In both pre and post treatment measurements, the differences tend to increase as the mean MTV increases. Variability (scatter) does not change significantly, while there is a linear trend in both plots, namely, differences increase as the mean MTDmax increases.

The differences between expert’s and CAD’s measurements of MTV and MTD_max_ were analyzed by Bland–Altman plots shown in Figure 3 and Figure 4, respectively [13]. The graphs also list the bias, i.e., the gap between the horizontal line at mean difference and the zero differences, and the lower and upper limits of the 95% confidence interval for the mean difference. Note that (a) the further the bias is from zero, the larger the mean difference and (b) the wider the limits of agreement, or disagreement, the more ambiguous the measurements are.

### 2.4. Weighted Kappa Measurements of Agreement

Linearly weighted Kappa was used to determine the agreement between the expert’s and CAD’s PET measurements and the clinical F/U, i.e., the classes shown in Figure 1 and Figure 2 [14,15]. Results are summarized in Table 4.

## 3. Discussion

This study aimed at addressing the following questions regarding HL and NHL imaged by ^18^F-FDG PET/CT:

1. How good therapy response predictors are PET standard metabolic measurements of MTDmax and SUVmax of lymphomas? To answer this, the MTDmax and SUVmax of 24 NHL and 21 HL patients were compared to the clinical outcome 6 months post treatment. Results showed that SUVmax had the highest agreement with the clinical outcome post treatment while the expert’s MTDmax measurements had moderate agreement. The CIs for both metrics were relatively wide due to the small sample size of our study, but the relative significance is not affected, even at the lower limit [15]. The CAD MTDmax measurements differed from the expert’s measurements and had poor correlation with the clinical follow-up. Differences for the larger size masses were often more than 100%, and this was puzzling considering that an expert also evaluated the CAD algorithm’s segmentation performance and deemed it acceptable. It should be noted, however, that the expert’s MTDmax and SUVmax values used in our analysis were recorded from the clinical diagnostic report, and the expert who did the formal clinical interpretation was different from the expert who participated in our segmentation process. It is well documented in several studies that a large margin is applied during standard clinical measurements while interobserver variability is high [1]. Finally, SUVmax was also measured by our algorithm, but these values were not reported here because they did not differ from the expert’s as they were both based on similar mathematical definitions [16].
2. How good are MTV measurements for the prognosis of the disease, and how do they compare to the standard measurements of MTDmax and SUVmax? Results showed that the expert’s MTV manual measurements have a fair agreement with the clinical follow-up. CAD MTV measurements showed a moderate agreement as is also indicated in other similar reports [17,18,19]. CAD’s better performance may be explained by the fact that CAD MTV values were based on more consistent ROI contours while the expert’s MTV values were based on rough elliptical contours around the tumor area in the various slices. CAD measurements were reproducible and faster compared to the expert and can be highly accurate, particularly when semi-automated, i.e., when initiated by an expert.
3. Is the MTAmax of any value? This parameter is rarely used or measured in studies of metabolic tumor size measurements. It showed fair agreement with the clinical outcome and its value was not considered significant.
4. Are there differences between HL and NHL cases? It seems that both the expert and the CAD performed better on the NHL than the HL masses. The NHL cases had masses with larger diameters and volumes than the HL but there was no indication, given our relatively small sample size, that the accuracy of measurements depended on the size.
5. How does the PET segmentation algorithm’s parameters affect measurements? The adaptive thresholding segmentation is a key element in our CAD approach, and the selection of a threshold impacts the final result. The 50% threshold was considered the optimum threshold for our algorithm. The selection was determined by a receiver operating characteristic (ROC) study, which was performed with five threshold values (30%, 40%, 50%, 60%, and 70%) on a subset of images where the masses where outlined by an expert and these outlines were considered “ground truth” [20]. The ROC analysis showed that a threshold of 50% yielded the best agreement, with the ground truth followed by the thresholds of 40% and 70%. To test it further, all three thresholds were used for the metabolic size measurements. Comparisons with the clinical outcome were conducted for all three sets of measurements. The 50% threshold yielded the best results and these are reported here.

How does metabolic tumor size compare to anatomic size, and is there any prognostic value in the latter? To address this question, we performed, as indicated above, similar 1D, 2D, and 3D size measurements of the HL and NHL masses in the corresponding CT images. A different segmentation algorithm was applied to CT than the one described in the following section for the PET images. The algorithm involved an initialization step based on wavelets and fuzzy C-means unsupervised clustering and a Markov Random Field step for final tumor segmentations [21,22]. CT measurements were significantly different from the PET measurements by either the expert or the CAD, as can be seen from Table 1, Table 2 and Table 3. They correlated poorly with the clinical outcome showing little, if any, prognostic value. CT images of lymphomas have poor contrast, making pre-processing a critical step in the segmentation process. Our CT results seem to agree with previous reports on the limited value of CT in assessing tumor response to treatment, and particularly lymphomas [2,12]. However, there may be some value in using CT as a guide to CAD methods for more accurate tumor segmentation on PET images. Considering also the rapidly advancing field of radiomics and its recent promising results on both solid and non-solid tumors, one could improve decision support for both HL and NHL by combining various CT and PET quantitative features, possibly including patient and clinical data [23,24]. Our conclusions are based on the selected segmentation methods and the expert. It is apparent from the literature that there is significant variability among observers and among processing methodologies. So, it is possible that an average measurement from multiple observers with different levels of expertise may alter the results and reduce potential biases and variances. Similarly, more advanced artificial intelligence algorithms may yield better and more accurate metrics with better correlation to clinical outcome. In addition, combinations of metrics from metabolic and anatomical data may lead to more powerful markers. One has to weigh, however, the computational load, the time to process, and the cost:benefit ratio of various automated or semi-automated approaches relative to the current clinical standard.

Finally, the omission of total lesion glycolysis (TLG) measurements of these masses may be considered a weakness of the study. TLG is generally considered a useful metabolic marker [25]. However, the estimation of TLG requires an accurate ROI outline in order to estimate a mean SUV that enters the TLG calculation. Considering the observed differences between expert and CAD on the ROI outlines and the small sample size of our study, we decided to exclude the TLG estimates from this work, as they strongly depend on the selected regions. A pilot work suggested that TLG may be of value in optimizing automated ROI segmentation, and this aspect is currently under investigation. In addition, the Deauville five point scale was not used as classification guide and it is possible that it may also impact the prognostic value of the metabolic metrics [26].

## 4. Materials and Methods

### 4.1. Patients and Data Coding

The demographics of the patients are listed in Table 5.

The database of the Nuclear Medicine Department of the Biomedical Research Foundation of the Academy of Athens was reviewed and serial PET/CT examinations were selected for the study that satisfied the following criteria:

(a) Patients should have one mass, non-operable, that underwent similar clinical treatment that included chemotherapy and radiation therapy.
(b) All patients should have at least two PET/CT examinations, one before (baseline) and one after treatment.
(c) All patients should have a clinical follow up 6 months after the end of their treatment and be classified according to the RECIL as in remission (positive response to treatment) with either complete or partial response with the tumor reduced in size or in relapse (negative response to treatment) with either no change in the tumor size or increase in size or appearance of new lesions.

Each mass was assessed by an expert nuclear medicine physician on the PET/CT images of each patient, and ground truth files were generated for each mass that included the maximum diameter pre and post treatment on the CT and PET images, and the SUVmax of each lesion pre and post treatment from the PET images following criteria used in the clinical practice. In addition, there was clinical follow of the patients one year after the end of treatment and cases were classified as remission or relapse according to RECIL.

### 4.2. ^18^F-FDG-PET/CT Imaging

A hybrid Biograph 5 PET/CT system (Siemens Healthcare GmbH, Erlangen, Germany) was used for imaging, pre and post-treatment. The same whole body imaging protocol was used pre and post treatment. All patients fasted for at least 6 h before the PET/CT study. The radiopharmaceutical was injected intravenously (370–555 MBq or 10–15 mCi) without contrast. Image acquisition started 1 h after intravenous administration at which time no patient had glucose level higher than 160 mg/dL. Patients were imaged in the supine position with their arms placed above their heads when possible. The acquisition time was 2–4 min per bed position. CT scans began at the orbitomeatal line and progressed to the upper thighs. CT images were acquired with 30 mA, 130 kV, axial slice thickness of 5 mm and table feed rotation of 27 mm per rotation. PET imaging followed immediately over the same body region. The CT data were used for attenuation correction and images were reconstructed using a standard ordered-subset expectation maximization algorithm. PET image reconstruction matrix size was 168 × 168 pixels with a voxel size of 4.06 mm × 4.06 mm × 2.5 mm [6].

### 4.3. Metabolic Parameter Measurements

**Figure 5:**
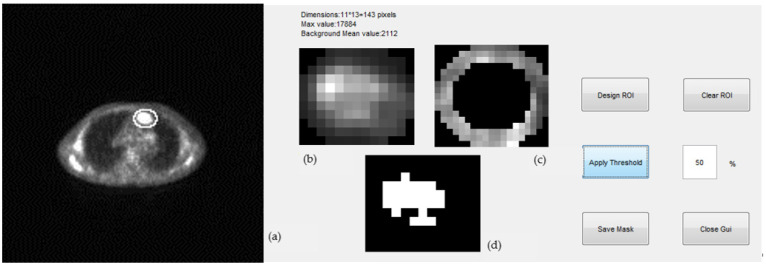
Representative segmentation of a mass from a PET slice where maximum diameter and area is measured using an in-house developed interface. (**a**) An ellipse was drawn around the tumor by an expert. (**b**) Area within the ellipse was shown in magnification to the expert for review. (**c**) 3-pixel ring was defined on the elliptical border for the calculation of the background intensity. (**d**) Final tumor ROI segmentation by the algorithm at a given threshold.

The metabolic size of the masses in 1D (maximum tumor diameter), 2D (maximum tumor area), and 3D (tumor volume) were determined by an in-house developed algorithm and user-interface in MATLAB R2013b. Tumors were first segmented on multiple slices using a semi-automated approach, a representative example of which is shown in Figure 5. The segmentation procedure included the following steps:

(1) Expert selected the PET and corresponding CT slice of a scan where tumor appeared at maximum diameter; we will refer to this as the “central” slice.
(2) An ellipse was drawn by the expert around the region of interest (ROI) by selecting “Design ROI” on the user-interface (Figure 5a). The expert was given the option to repeat this step if the result was not satisfactory as presented in Figure 5b by selecting “Clear ROI”.
(3) A background ring was defined automatically on the border of the elliptical region drawn by the expert. The ring was 3 pixels wide and its mean pixel value was used for the estimation of the threshold of the segmentation (Figure 5c). Pixels with values greater than a selected percentage of the mean background value were considered as part of the tumor, otherwise they were rejected. A non uniform region was finally defined for a given threshold as the tumor ROI and was used for the estimation of the tumor maximum diameter (mm) and tumor maximum area (mm^2^) ((Figure 5d).
(4) The “central” slice elliptical contour of the expert was automatically projected by the algorithm to slices above and below where tumor appeared. This number ranged from 5–20 slices per case depending on the tumor size.
(5) Same ROI segmentation process of step (3) was applied to all slices and for three different thresholds, 40%, 50%, and 70%.
(6) The MTV was estimated by adding all 2D ROIs and using the voxel dimensions.

Measurements of MTDmax, SUVmax, and MTV were also performed manually by a nuclear medicine expert using Biograph’s user interface standard tools. For the volume measurements, the expert outlined a tight ellipse around the tumor region in all PET slices where the tumor was observed and the volume was estimated in mm^3^ by summing all the pixels in the outlined areas and multiplying with the voxel size.

### 4.4. Statistical Analysis

The analysis of the data used descriptive statistics, Bland–Altman plots for testing the agreement of the sets of measurements, and weighted Cohen’s kappa to test the agreement of the various parameters with the clinical outcome, which may be considered as their prognostic value.

The Bland–Altman plots graphically demonstrate the difference between the expert and CAD estimates of the measured parameters as a function of their mean values [13]. The three horizontal lines are drawn at the mean difference, and at the limits of the agreement, which are defined as mean difference ±1.96 SD of the differences. The weighted Cohen’s kappa was used to measure the agreement between the various metrics and the clinical outcome, because differences were ranked and were not considered to be equally important [14].

## 5. Conclusions

The hypothesis of our study was that 3D metabolic parameters are key predictors of response to treatment and would significantly overpower 1D metrics. We tested our hypothesis on HL and NHL cases and concluded that computer assisted MTV measurements have the potential to be a useful marker for treatment response, but do not differ significantly from MTDmax while they fall short of SUVmax. It is more likely that combinations of various metabolic, anatomic, and imaging parameters will yield better prognostic markers. Given the additional load and variability of radiomics measurements, the use of fast, standardized, and reproducible CAD tools in clinical PET/CT practice seems inevitable.

## Figures and Tables

**Table 1:** Average PET metabolic parameter measurements and standard deviations (in parentheses) pre and post treatment for the HL cases.

HL Parameter	Expert		CAD	
	Pre	Post	Pre	Post
MTDmax (mm)	18 (9)	16 (9)	10 (3)	10 (2)
MTAmax (mm^2^)			1181 (597)	1060 (278)
MTV (mm^3^)	8557 (9718)	12,485 (14,370)	7507 (4216)	8845 (6410)
SUVmax	7 (4)	6 (3)		

**Table 2:** Average PET metabolic parameter measurements and standard deviations (in parentheses) pre and post treatment for the NHL cases.

NHL Parameter	Expert		CAD	
	Pre	Post	Pre	Post
MTDmax (mm)	28 (22)	26 (23)	11 (6)	11 (7)
MTAmax (mm^2^)			1768 (2283)	2070 (3403)
MTV (mm^3^)	52,884 (188,602)	17,688 (22,625)	50,621 (149,033)	19,167 (27,691)
SUVmax	10 (8)	8 (6)		

**Table 3:** Average automated CT size measurements and standard deviations (in parentheses) pre and post treatment for the HL and NHL cases.

NHL Parameter	HL		NHL	
	Pre	Post	Pre	Post
MTDmax (mm)	20 (8)	24 (10)	34 (37)	26 (24)
MTAmax (mm^2^)	167 (130)	235 (161)	740 (1410)	478 (987)
MTV (mm^3^)	9284 (10,876)	15,034 (15,348)	160,370 (402,619)	22,943 (53,945)

**Table 4:** Weighted Cohen Kappa statistic for the agreement between the expert’s and CAD’s classification of patients based on changes in metabolic parameters and the clinical outcome; linear weighting was used. The type of agreement suggested by the kappa value and the 95% confidence interval (CI) is also listed.

Pair	Weighted k-Value	Agreement	95% CI
Expert % change in MTDmax—Clinical F/U	0.47	Moderate	(0.25,0.70)
CAD %change in MTDmax—Clinical F/U	0.18	Slight	(−0.19,0.54)
CAD %change in MTAmax—Clinical F/U	0.34	Fair	(0.09,0.60)
CAD % change in MTV—Clinical F/U	0.52	Moderate	(0.30,0.70)

**Table 5:** Demographics of the 24 NHL and 21 HL patients. Data are presented as mean ± standard deviation and as numbers. Age was recorded at the time of the first PET/CT scan. *p*-values were estimated by the two-tailed student *t*-test and the chi-square test for the gender numbers.

Parameter	NHL	HL	*p*-Value
Age (yr)	48.5 ± 17.7	43.5 ± 19.2	0.38
Weight (kg)	77.0 ± 11.9	78.7 ± 5.9	0.49
Height (cm)	171.5 ± 7.1	169.9 ± 8.9	0.51
Gender (Male/Female)	16/8	8/13	0.06

## Data Availability

Data of this study are not publicly available according to IRB approval. More information may be provided upon request to the corresponding author.
